# Recognition of Spatial Distribution of CNT and Graphene in Hybrid Structure by Mapping with Coherent Anti-Stokes Raman Microscopy

**DOI:** 10.1186/s11671-020-3264-8

**Published:** 2020-02-07

**Authors:** Alesia Paddubskaya, Danielis Rutkauskas, Renata Karpicz, Galina Dovbeshko, Nadezhda Nebogatikova, Irina Antonova, Andrej Dementjev

**Affiliations:** 1 Center for Physical Sciences and Technology, Sauletekio ave. 3, LT-10257 Vilnius, Lithuania; 2 Institute for Nuclear Problems, Belarusian State University, 220006 Minsk, Belarus; 3 Institute of Physics, National Academy of Sciences of Ukraine, 46 Nauki Ave, Kyiv, 03680 Ukraine; 4 Novosibirsk State University, Pirogov st. 2, Novosibirsk, Russia 630090; 5 Rzhanov Institute of Semiconductor Physics SB RAS, Lavrentiev av.13, Novosibirsk, Russia 630090

**Keywords:** Graphene, CNTs, CARS imaging, G-band

## Abstract

The shape of coherent anti-Stokes Raman scattering (CARS) spectral line depends on the ratio of the vibrational and electronic contributions to the third-order susceptibility of the material. The G-mode (1590 cm^−1^) of graphene and carbon nanotubes (CNTs) exhibits opposite features in the CARS spectrum, showing “dip” and “peak,” respectively. Here, we consider the CARS spectra of graphene and carbon nanotubes in terms of Fano formalism describing the line shapes of CARS resonances. We show that imaging at only 1590 cm^−1^ is not sufficient to separate the constituents of a composite material consisting of both graphene and CNTs. We propose an algorithm to map the graphene and CNTs in a composite material.

## Introduction

In the recent years, the composites or hybrid materials based on graphene and carbon nanotube (CNT) have become a subject of extensive studies since synergistic effects of such kind of combination allowed for a significant progress in the development of novel flexible transparent electrodes [1–3], supercapacitors [4, 5], and sensitive biological sensors [6]. It was demonstrated, for example, that in a polymer composite the presence of CNTs prevented aggregation of graphene nanoparticles and, on the other hand, graphene nanoparticles improved the dispersion of CNTs [7, 8]. That enhanced the total dc conductivity and improved the mechanical and electromagnetic shielding interface properties of CNT/graphene-based composite [9, 10]. In Ref. [3, 11], it was shown that the presence of a small number of CNTs on the surface of chemical-vapor-deposited (CVD) graphene results in a significant decrease of the sheet resistance, keeping the optical transmittance at the same level.

Significant progress has been achieved in the development of various techniques for the synthesis of CNT/graphene hybrid structures and composites. At the same time, it is often desirable to be able to map the spatial distribution of the constituents. Despite the attempts to use the optical microscopic fluorescence or Raman scattering imaging, it is still a challenging problem [12].

Raman spectroscopy is a powerful tool to characterize carbon material and its composites [13, 14]. However, intrinsically weak Raman signal results in prohibitively long acquisition times that precluded the possibility to image the carbon material in the biological samples and polymer matrixes [12]. Long imaging times also limited the possibility to analyze the CNT distribution on the graphene surface at a spatial scale of several microns.

Due to the unique graphene band structure, photons of any energy are in resonance with real electronic states. It leads to a very strong nonlinear optical response and can be used for high-contrast imaging of graphene flakes consisting of a single or a few layers [14]. In this context, as an alternative approach, the coherent analog of spontaneous Raman scattering or coherent anti-Stokes Raman scattering (CARS)—a particular case of four-wave mixing—can be applied to characterize CNTs and/or graphene [14, 15]. Moreover, the coherent nature of CARS provides an opportunity to enhance significantly the obtained signal thus allowing the fast imaging with pixel acquisition time up to several microseconds [16]. It is worth noting that the main contribution to the CARS spectra of graphene comes from the electronically enhanced nonresonant background. At the same time, the contribution of the vibrational component to the four-wave mixing seems to be much smaller than the electronic one. Due to the Fano resonance nature [17], in this case at the resonance frequency, a “dip” instead of a “peak” should appear in the CARS spectrum. This prediction is confirmed by the previously obtained CARS spectra of graphene, where a “dip” in the form of antiresonance was observed at the G-mode frequency (1590 cm^−1^) [18]. The first theoretical explanation of the physical mechanism responsible for the CARS signal of single- and few-layer graphene has only recently been described in details in Ref. [19]. Using time-delayed FWM (four-wave mixing) technique, the authors also experimentally demonstrate how the inter-pulse delay, ∆*T*, can be used to modify the G-mode peak profile.

On the other hand, as it was shown in our previous work [20], for CNTs, the vibrational contribution to the third-order susceptibility prevails over the electronic contribution, and the spectrum at the G-mode frequency reveals Raman-like peak.

Thus, the CARS spectra of graphene and CNTs are drastically different in the area of the G-band, and this can be used for their identification in a composite. To our knowledge, investigation of a composite consisting of materials with opposite spectral features at the same resonance frequency using CARS microscopy has not yet been carried out.

In this work, we provide the systematic analysis of the possibility to separate tiny amounts of CNTs deposited on the surface of CVD graphene by CARS spectroscopy. Furthermore, we propose the mapping algorithm which can be used for future characterization of CNT/graphene hybrid systems.

## Methods

### Sample Preparation

The graphene films or single-layer graphene (SLG) used in our experiments was synthesized on 25-μm-thick copper foil (99.9%, Alfa Aesar) by CVD in a hot wall tube furnace (Carbolite Gero, 30–3000 °C). First, the piece of copper foil was loaded into a horizontal furnace and all system was evacuated down to 0.06–0.1 mBar. After that, the system was heated up to 1050 °C in hydrogen atmosphere at 2 mBar with 60 sccm flow. To smooth the substrate surface, as well as to reduce the native copper oxide and other impurities on the surface, the copper was additionally annealed for 1 h at 1050 °C. After that, to grow graphene, methane was introduced into the system for 30 min. In our experiments, the molar ratio of hydrogen and methane was set to 2:1, and the total pressure was ~ 5 mBar. After growth, the system was cooled down to room temperature in static hydrogen atmosphere (total pressure was around 2 mBar). The multi-layer graphene (MLG) film was grown identically but the time of methane incubation was increased.

### Characterization Methods

For subsequent characterization, the obtained graphene film was transferred on a dielectric substrate using the technique reported in [21]. A polymethyl methacrylate (PMMA) solution was spincoated on a 1 cm × 1 cm graphene/copper bilayer and then baked at 60–100 °C for 30 min. After that, the copper substrate was etched with FeCl_3_ solution and the obtained “free-standing” graphene/PMMA film was washed several times with deionized water and collected on a 0.17-mm-thick glass coverslip. The PMMA was next removed with acetone.

**Fig. 1:**
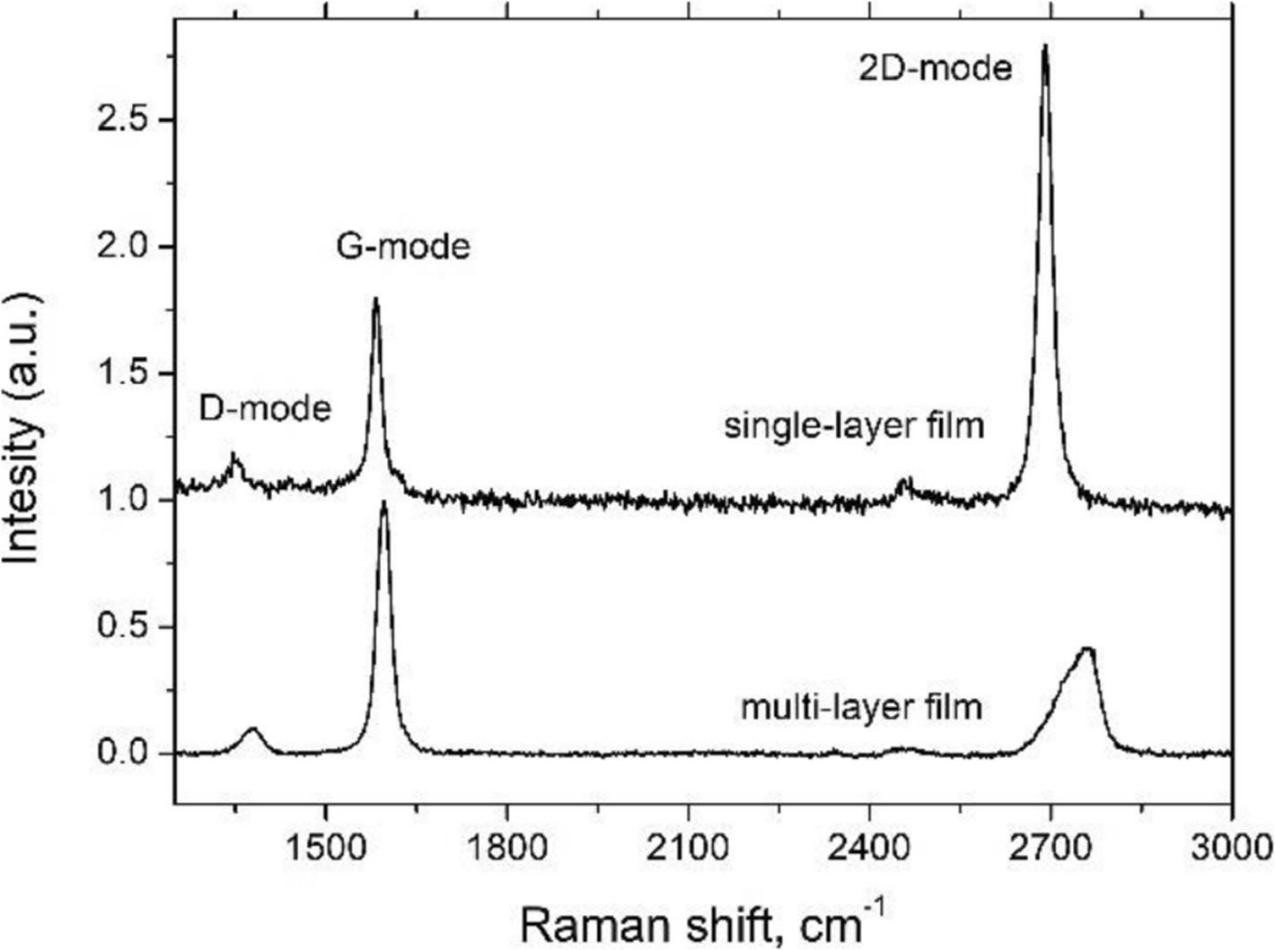
Raman spectra of SLG and MLG carbon films transferred on a glass substrate

The quality of transferred graphene films was assessed with Raman spectroscopy. The measurements were carried out at room temperature using a confocal Raman spectrometer equipped with a 600 lines/mm grating and 200-μW, 532-nm excitation laser. All spectra were collected using a × 100 objective, and to avoid sample degradation, the exposition time was set to 30 s. Figure 1 compares the typical Raman spectra of SLG and MLG obtained in our experiments. One can see that the two most prominent spectral features typical for carbon materials, the G-band at ~ 1586–1596 cm^−1^ and the 2D-band at ~ 2700 cm^−1^, are present in the spectra of both SLG and MLG films. Moreover, in the case of SLG, the 2D-mode exhibits a single, sharp (full width at half maximum, FWHM, ~ 30 cm^−1^), and symmetric peak which is two times more intense than the peak of the G-mode. On the other hand, in the case of MLG, the shape of the 2D-mode is asymmetric and consists of two components, indicating the multilayered structure. It is worth noting that the low intensity of the D-mode (~ 1360 cm^−1^) for both samples indicates the presence of the significant number of defects in the structures.

To make graphene/CNT system, we used single-walled carbon nanotube (SWCNT), Inc., SG65i from Sigma-Aldrich. The hybrid samples were prepared by depositing the SWCNT powder on the surface of graphene films transferred to the glass coverslip.

Home-built CARS system described previously [22] was used for the CARS imaging. Briefly, the Olympus IX71 microscope combined with the dual-wavelength 1-MHz picosecond laser source (EKSPLA Ltd.) and a piezo scanning system (P-517.3CL, Physik Instrumente GmbH & Co) was utilized for raster scanning of the sample. The exciting light was focused on the sample with an oil-immersion objective (Olympus, Plan Apochrom., 60X, NA 1.42). The CARS signal was detected with the avalanche photodiode (SPCM-AQRH-14, Perkin Elmer), connected to a multifunctional PCI board (7833R, National Instruments). The fundamental wavelength (1064 nm) and tunable-wavelength radiation from the optical parametric generator (OPG) were used as Stokes (*ω*_S_) and pump (*ω*_p_) excitation beams, respectively. The fingerprint region was studied in the range from 1250 to 1700 cm^−1^. For this, the OPG was tuned from 938 to 900 nm and the resulting CARS signal (*ω*_AS_ = 2*ω*_p_ − *ω*_S_) from 840 to 782 nm was detected. Long-pass (cutoff at 860 nm) and short-pass (cutoff at 780 nm) filters were applied to spectrally separate the CARS signal in the epi-detection scheme. Excitation powers of 10–50 μW and 50 μW were employed for the pump and Stokes beams, respectively.

## Results and Discussion

**Fig. 2:**
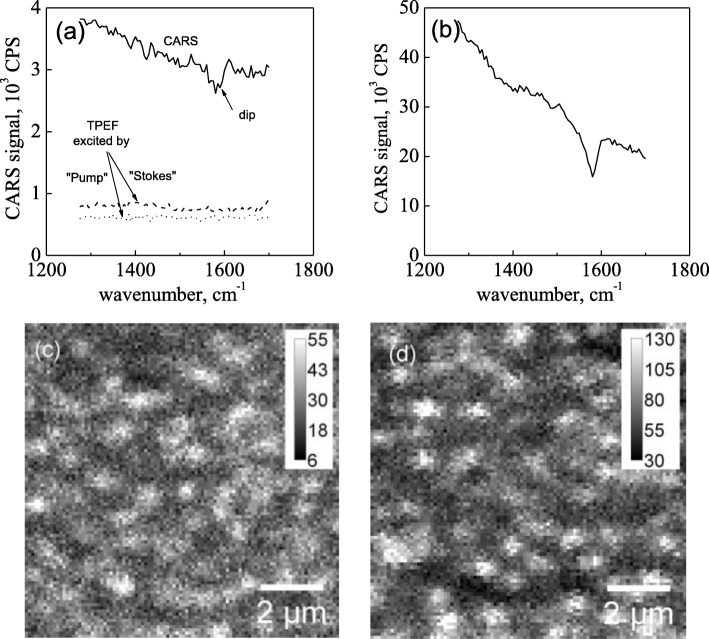
**a** TPEF from the pump (dashed line) and from the Stokes (dotted line) beams, both contribute to the total CARS signal (solid line) within the fingerprint range. Despite the TPEF background, the noticeable “dip” at 1585 cm^−1^ (Exc: pump 30 μW/Stokes 100 μW) is well seen in the CARS spectrum of SLG. The dip at the G-band frequency is clearly manifested in the spectrum of MLG. **b** The contribution of TPEF to the background (~ 50% of amplitude) was the same for single-layer and multi-layer graphene. CARS images of SLG and MLG respectively recorded at 1585 cm^−1^ (Exc: pump 310 μW/Stokes 530 μW) are presented in **c** and **d**

It is known that single-layer graphene produces a complex CARS response. In addition to the CARS photon with energy of 2*ω*_p_ − *ω*_s_, in the sample, a broadband two-photon-excited fluorescence (TPEF) originating from both Stokes and pump excitation beams is also generated (see Fig. 2a). Note that the presence of the TPEF reduces the ability of the CARS spectroscopy for graphene characterization. However, it is easy to show that the contribution of the TPEF to the total detected signal can be substantially reduced (up to 40%) by varying the intensities of the Stokes and the pump beams. The CARS spectrum of SLG is presented in Fig. 2a. One can see that a small “dip” at the G-band frequency is clearly observed, and it indicates that the contribution of the nonresonant component to the CARS response is dominant [17, 21]. Figure 2c demonstrates the CARS image of graphene obtained at the frequency of the G-band. In fact, the nature of the bright spots and the dark areas is not completely clear. Most probably, such spots are the defect-induced luminescence centers. On the other hand, due to the linear polarization of both excitation beams, the efficiency of the CARS generation should depend on the roughness of the graphene surface. Moreover, since the contribution of the TPEF and CARS to the total signal is almost equal, both mechanisms may be responsible for the variable brightness of the graphene sheet in the image.

Multi-layer graphene (~ 10 layers) showed the same “island” structure (Fig. 2d). Despite the fact that an increase of the number of graphene layers smoothes the total signal and as a result leads to uniform picture, the interpretation of the bright spots in the case of MLG is at the moment unclear. It is also worth noting that increasing the number of graphene layers leads to improvement of the signal-to-noise ratio and as a result improves “dip” contrast (CARS contribution to total signal grows faster than TPEF). However, at present, the dependence of the “dip” depth on the number of graphene layers as well as the absence of the quadratic dependence of the observed CARS signal vs the amount of graphene layers [14] is still unclear and should be investigated separately which is beyond the framework of this work.

It is known that the CARS signal is a product of the interference of resonant and nonresonant processes. In other words, a vibrational discrete resonant signal interferes with an electronic continuous nonresonant signal. The overlap of discrete and continuous spectra appears as asymmetric profile in the spectral band and is well described by Fano formalism [17, 23, 24]. The Fano formula (1) contains an asymmetry parameter *q* describing the relationship of the resonance and nonresonance contributions. In expression (1), *E* is a difference between the photon energies of the pump and the Stokes beams, *Ω* is the vibrational resonance energy, and *Γ* is the width of the resonance line.

1 $$ {I}_{\mathrm{CARS}}=A\frac{{\left[\left(\Omega -E\right)+\Gamma q\right]}^2}{{\left(\Omega -E\right)}^2+{\Gamma}^2} $$

When nonresonance prevails over resonance, then |*q*| ≪ 1 and the lineshape is a symmetric “dip” [17]. In CARS, the *q* parameter is defined as the ratio of the resonant and nonresonant parts of the third-order susceptibility. For graphene, we have a limiting case of Fano resonance, where the nonresonant contribution (continuous spectrum) is much larger than the resonant contribution (discrete spectrum). Thus, the “dip” obtained in the graphene spectrum at the resonance frequency indicates the electronic nature of its CARS response.

At the same time, as it was previously shown in [20], the remarkable “peak” is observed in the CARS spectrum of the CNTs at the frequency of the G-band. Moreover, in the case of semiconducting CNTs with 1.1 nm diameter, due to the triple resonance, the CARS signal can be significantly enhanced, which allows to detect the CARS response from individual CNTs or their small agglomerates. It is worth noting that CARS enhancement and the appearance of the Raman-like profile occur only for SWCNTs of a certain diameter, for which the arrangement of the discrete energy levels is in resonance with the energy of the incoming excitation photons.

**Fig. 3:**
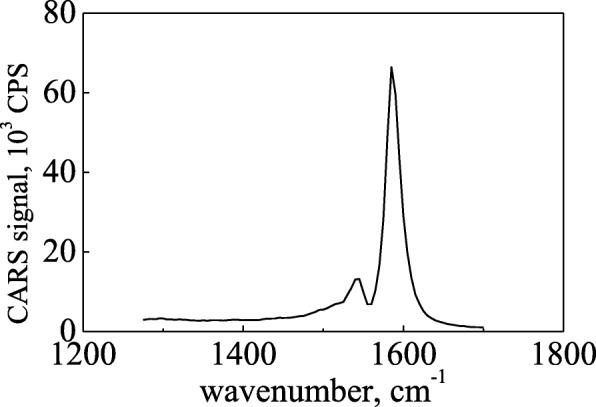
Typical CARS spectrum of CNTs (SWCNT, Inc., SG65i from Sigma-Aldrich) with Raman-like lineshape

With the diameter of the probed CNTs in our experimental setup, the resonance conditions were fulfilled showing both a strong CARS response and a Raman-like profile of the G-band (Fig. 3). In context of the Fano formalism, it means that the asymmetry parameter |*q*| ≫ 1, and hence, the shape of the G-band is close to Lorentzian [17].

To exploit the observed difference in the shape of the G-band resonance, the study of the graphene/CNT system by the CARS technique requires a suitable criterion for the separation of these carbon components. The imaging of such a composite system at the frequency of the G-band is not selective and associated analysis is problematic.

**Fig. 4:**
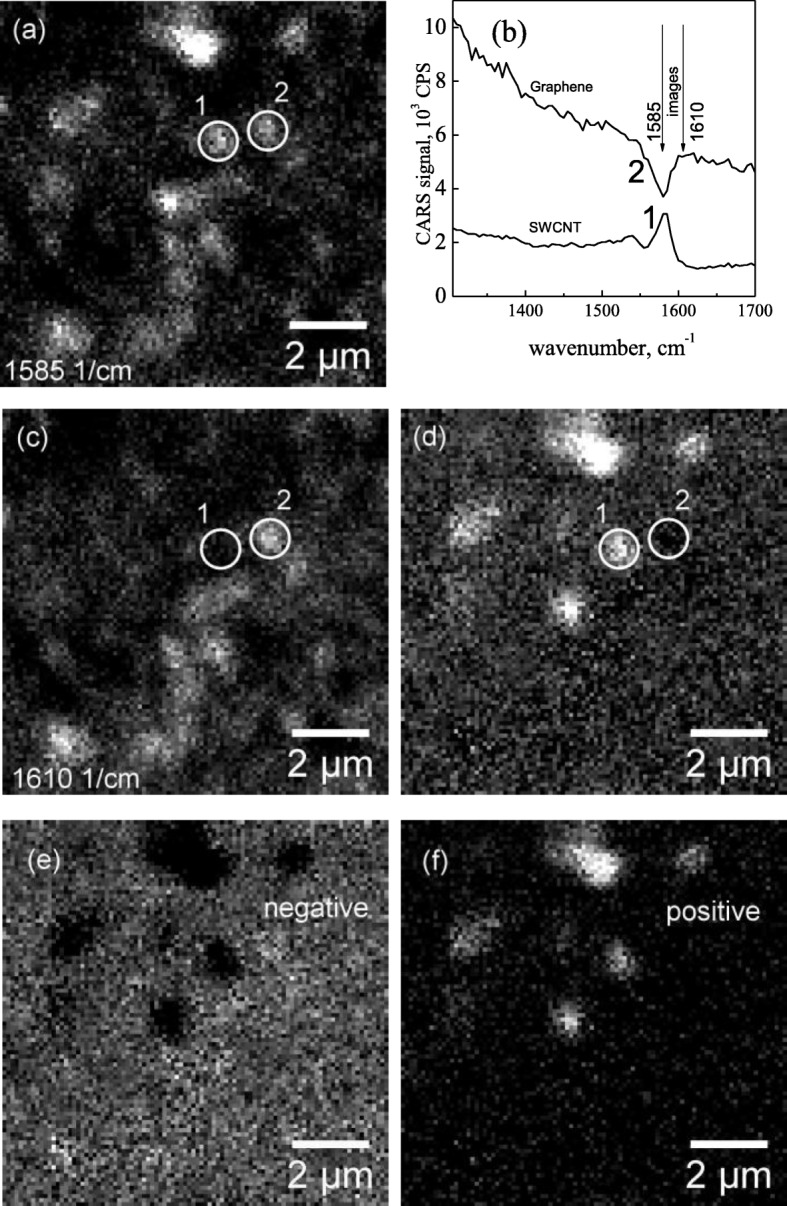
**a** Image of a CNT/graphene system obtained at 1585 cm^−1^. Point no. 1 and point no. 2 (the same areas on **a**, **c**, and **d** are circled and numbered) have the same brightness while corresponding spectra (**b**) at the resonance frequency show “peak” and “dip,” respectively. **c** Image of a CNT/graphene system obtained at 1610 cm^−1^. **d** The difference image of images **a** and **c**. After subtraction procedure separation of negative (**e**) and positive (**f**) amplitudes reveal graphene and CNTs respectively (see text). Brighter pixels in the pictures (**e**, **f**) correspond to a larger amplitude

Figure 4a shows the image of the CNT/graphene composite system recorded at 1585 cm^−1^. Some bright spots could be assigned to graphene forming a pattern similar to that shown in Fig. 2. At the same time, other bright spots were attributed to CNTs. The CARS spectra collected from two different points of similar brightness, point no. 1 and point no. 2, are presented in Fig. 4b. As can be seen, at the frequency of the G-mode, there is a “peak” for point no. 1 and a “dip” for point no. 2. However, the maximum amplitude of “peak” is approximately equal to the minimum of the “dip” (Fig. 4b). This means that, in practice, because both of those objects have the same brightness, the additional information is required for their separation. Figure 4c shows the imaging of the same area recorded at 1610 cm^−1^. As can be seen, some bright spots are not present, including the point no. 1. Because in the case of the CNTs the shift from 1585 to 1610 cm^−1^ should lead to the decrease of the signal, it is reasonable to assume that the spots that disappeared at 1610 cm^−1^ correspond to the tubes. Consequently, the objects remaining in the image at 1610 cm^−1^ correspond to the graphene. In other words, graphene can be efficiently separated from CNTs by mapping at any frequency away from the resonance (1585 ± 15 cm^−1^). According to our observations, to obtain the spatial distribution of the CNTs, it is useful to generate a pseudo-image based on the difference between the images acquired at 1585 and 1610 cm^−1^. Figure 4d demonstrates the image obtained by pixel-to-pixel subtraction of the data presented in Fig. 4a and c. One can see the CNTs appear as bright spots (point no. 1, the difference between the CARS signal at 1585 cm^−1^ and 1610 cm^−1^ has positive sing) while the signal from graphene is absent (point no. 2, the difference between the CARS signal at 1585 cm^−1^ and at 1610 cm^−1^ has a negative value). In general, the sign of difference between the CARS signal at 1585 cm^−1^ and at 1610 cm^−1^ can be used as one of the criteria to generate the images representing CNT (Fig. 4f) distribution and pure graphene area (Fig. 4e), respectively.

It is worth noting that there are other possibilities for the separation of graphene from CNTs by imaging. For example, it is possible to use the difference in fluorescence. Graphene has a noticeable TPEF, while the CNTs do not fluoresce. However, for CNTs of other diameters, which have not been studied in this work, the TPEF may arise, and then the use of fluorescence, as a contrasting mechanism, becomes more complicated. The study of other contrast mechanisms or their combination is beyond the scope of this article.

## Conclusions

In conclusion, the “peak” and the “dip” for SWCNT and graphene, respectively, observed at the resonance frequency of the G-band complicate their separation in imaging using CARS spectroscopy. This stimulates the search of an algorithm enabling the separation of the components in CNT/graphene composite system. The imaging only at 1585 cm^−1^ does not allow to separate the components. We have demonstrated that two images are necessary for this. While imaging at 1610 cm^−1^ gives direct mapping of graphene revealing its specific pattern, identification of CNTs requires images at both frequencies. The difference image obtained by subtracting the image at 1610 cm^−1^ from the image at 1585 cm^−1^ shows the distribution of CNTs. This approach allows separate imaging of CNTs and graphene with CARS microscopy and can be useful for future characterization of novel hybrid composite materials.

## Data Availability

The authors declare that the materials and data are available to the readers, and all conclusions made in this manuscript are based on the data which are all presented and shown in this paper.

## Abbreviations

CARS: Coherent anti-Stokes Raman scattering
CNT: Carbon nanotube
CNTs: Carbon nanotubes
CVD: Chemical-vapor-deposited
FWM: Four-wave mixing
MLG: Multi-layer graphene
OPG: Optical parametric generator
PMMA: Polymethyl methacrylate
SLG: Single-layer graphene
SWCNT: Single-walled carbon nanotube
TPEF: Two-photon-excited fluorescence
